# The different features of angiographic peri‐stent contrast staining after implantation of sirolimus‐eluting stents

**DOI:** 10.1002/ccr3.827

**Published:** 2017-02-08

**Authors:** Michiya Kageyama, Shichiro Abe, Iwamatsu Koichi, Hiroaki Nishida, Satoshi Kiozumi, Takahisa Nasuno, Shuichi Yoneda, Masashi Sakuma, Teruo Inoue

**Affiliations:** ^1^ The Department of Cardiovascular MedicineSchool of MedicineDokkyo Medical UniversityTochigiJapan

**Keywords:** Drug‐eluting stent, incomplete stent apposition, multiple interstrut hollow, peri‐strut staining

## Abstract

If we had a case with angiographic peri‐stent contrast staining(PSS)s after the first‐generation sirolimus‐eluting stent, we need a further observation using coronary imaging modalities to evaluate the risk of very late stent thrombosis due to PSSs and to continue or to resume the dual antiplatelet therapy if necessary.

## Introduction

Although the first‐generation drug‐eluting stent (DES), sirolimus‐eluting stent (SES), substantially reduced late loss, and thus, restenosis rate, novel issues such as a risk of very late stent thrombosis (VLST) have arisen 1. Therefore, it is not established how long we should continue a dual antiplatelet therapy (DAPT) after implantation of SES stent. In patients, who developed VLST, coronary angiogram often demonstrates peri‐stent contrast staining (PSS) after thrombus aspiration 2. Recent advances in imaging modalities including optical coherence tomography (OCT) 3 and coronary angioscopy 4 have provided us some novel information about angiographic PSSs. Now in this study, we report a case, who had a history of SESs stent implantation in both left anterior descending artery (LAD) and left circumflex artery (LCX), developed VLST at the SES‐stented site in the LAD alone 9 years after stenting. After thrombus aspiration, we carefully observed the stented sites using OCT and coronary angioscopy.

## Case Report

**Figure 1:**
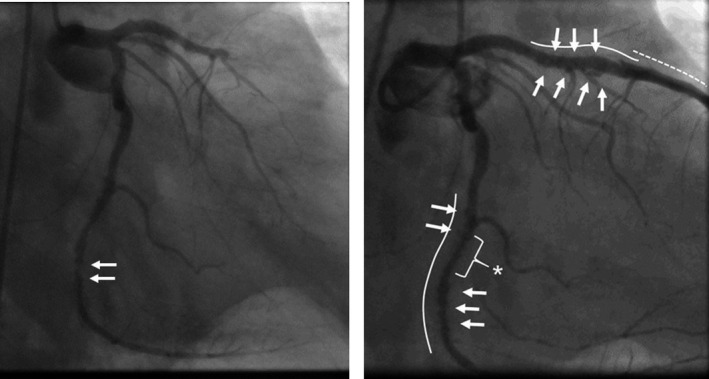
Emergent coronary angiography (30° right anterior oblique view) at acute phase of very late stent thrombosis (left). Total occlusion at one SES site of the LAD and in‐stent restenosis with 75% luminal narrowing at another SES site of the LCX (arrows) were shown. Coronary angiographic finding (30° right anterior oblique view) 20 days after admission (right). Solid lines indicate the sites of sirolimus‐eluting stent (SES) implanted 9 years ago, and dotted line indicates the site of bare metal stent (BMS) implanted latest. The PSS of irregular segmental type is evident at both SES sites of left anterior descending artery (LAD) and left circumflex artery (LCX) (arrows). The SES site of LCX showed also in‐stent restenosis (ISR) with 75% luminal narrowing (asterisk).

**Figure 2:**
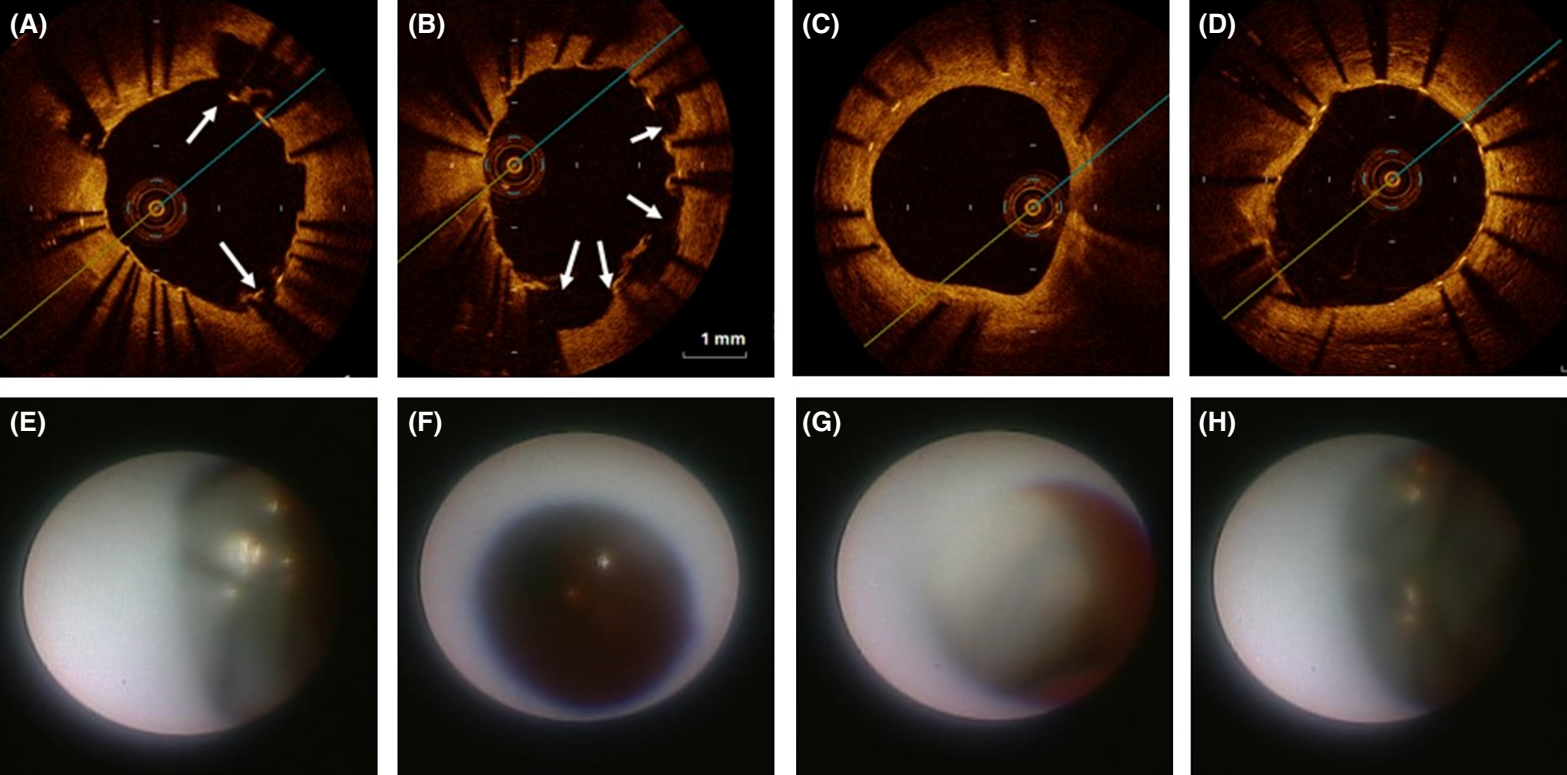
Findings of optical coherence tomography (OCT) (upper) and coronary angioscopy (lower) for the LAD. Incomplete stent apposition (ISA) at mid‐ (A) to distal SES site (B) (arrows), where the PSSs were angiographically evident by the OCT findings. Coronary angioscopic finding at this site showed that neointimal coverage over the stent struts was absent, but the neointima around the struts was white‐colored (E, F). At proximal SES site, where the PSSs were absent, OCT (C) and coronary angioscopy (G) showed that stent struts were covered by white‐colored homogeneous neointima. At the site of BMS newly implanted in the LAD at admission, stent struts were naked but stent apposition was complete by OCT (D) and coronary angioscopy (H).

**Figure 3:**
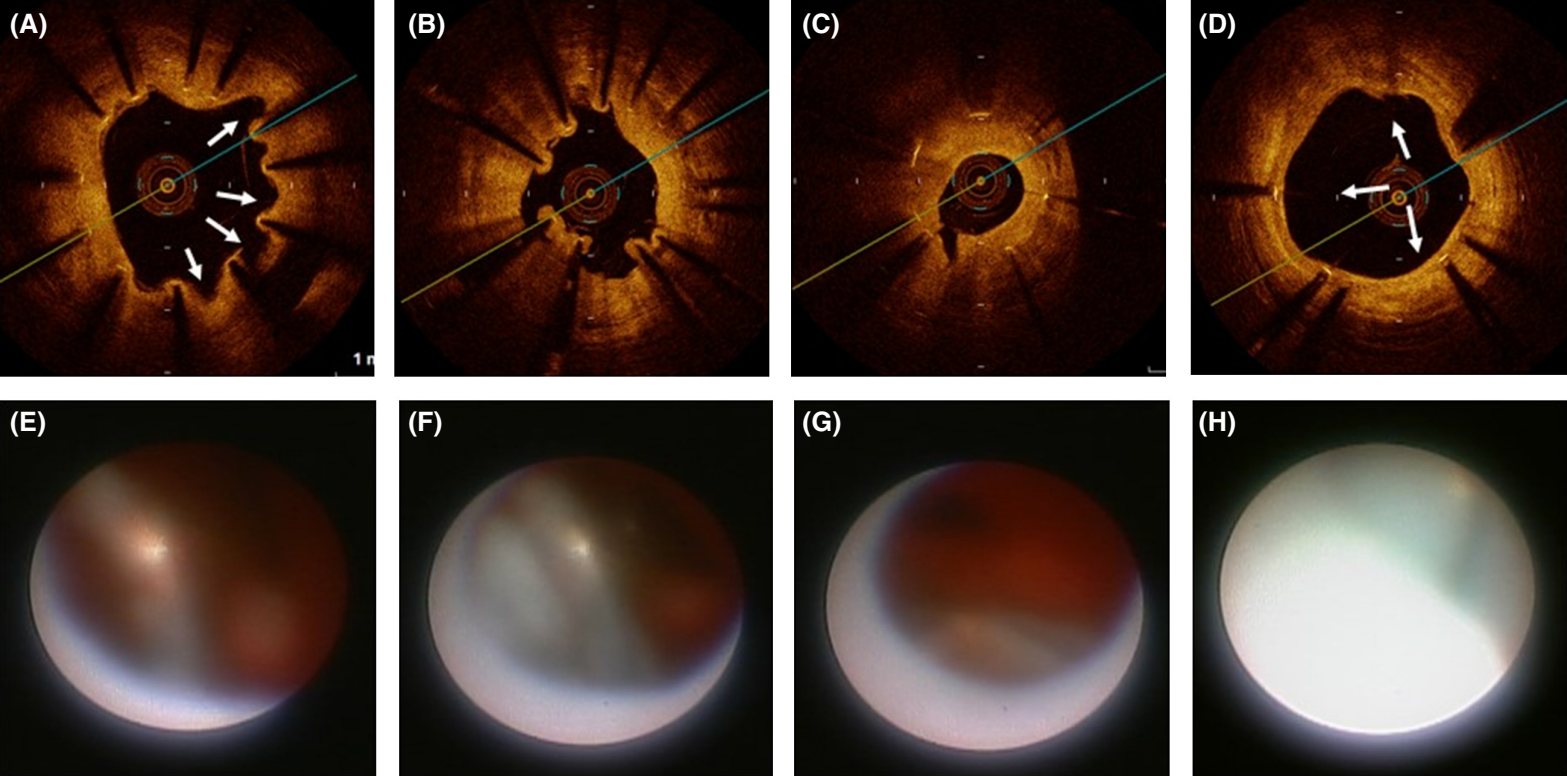
Findings of OCT (upper) and coronary angioscopy (lower) for the LCX. At the SES site, where the ISR was present but the PSSs were evident, multiple interstrut hollows (MIH) were seen by the OCT observation (A, B) (arrows). However, the stent struts between MIH were sufficiently covered with homogenous white‐colored neointima in OCT (A, B) and coronary angioscopy (E, F). At the most stenotic site, stent struts were covered with thick homogenous neointima (C, G). At the SES site, where the PSSs were absent, the MIH was absent and the stent struts were covered by white‐colored neointima in both OCT (D) and coronary angioscopy (H).

**Figure 4:**
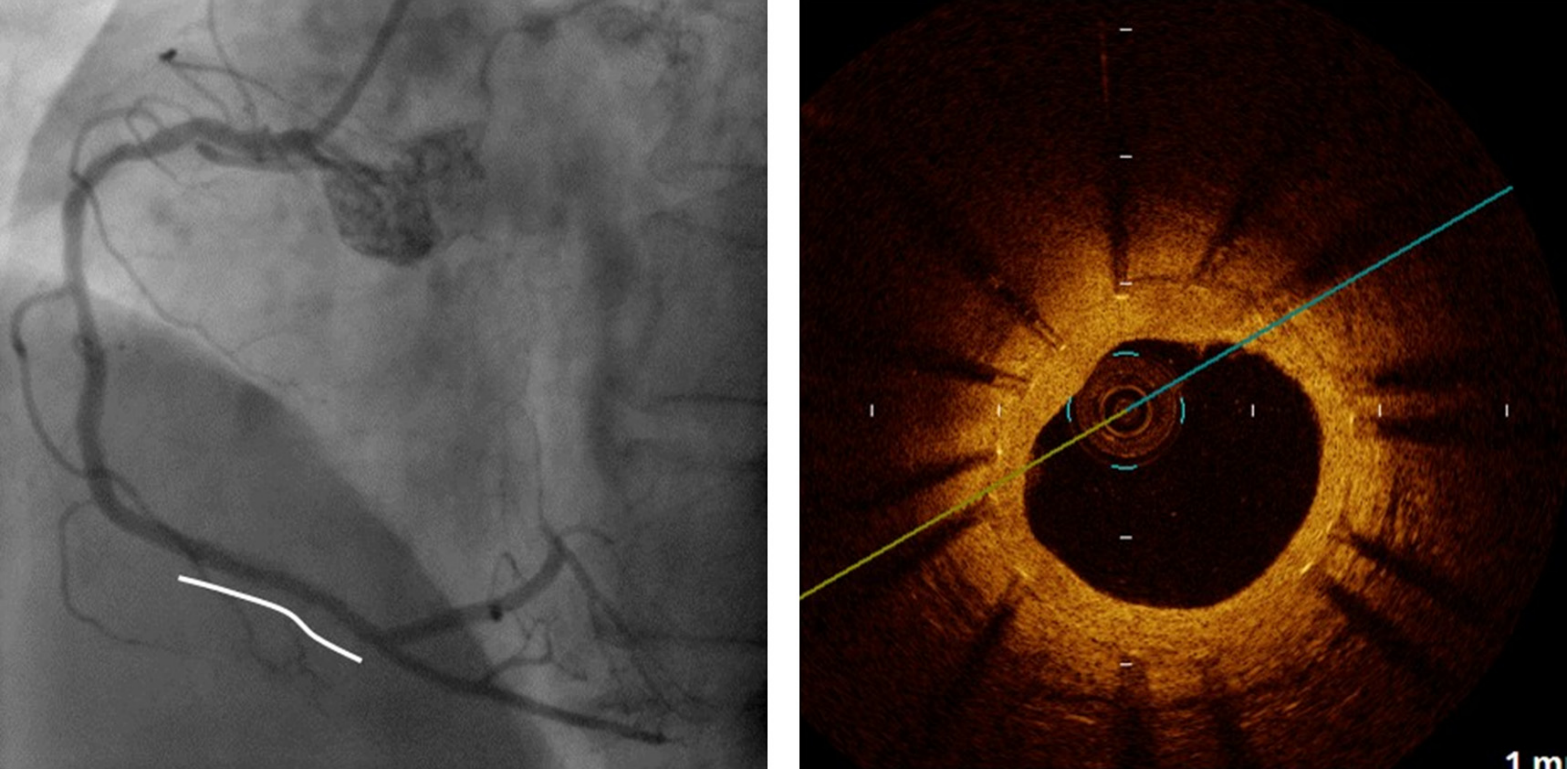
Findings of coronary angiography (left) and OCT (right) for RCA. At the BMS site (solid line) in the distal RCA, where the stent was implanted 13 years ago, the stent struts were covered by homogeneous high‐intensity neointima.

A 76‐year‐old male patient with acute ST‐elevation myocardial infarction was transported to emergency center of our hospital by ambulance. He had history of stent implantations with a bare metal stent (BMS) at distal right coronary artery (RCA) 13 years ago and that with two SESs at each of proximal LAD and mid‐LCX 9 years ago in another hospital. He had been taken 100 mg aspirin as a poststent antiplatelet therapy till admission. Emergent coronary angiography showed total occlusion at one SES site of the LAD and in‐stent restenosis (ISR) with 75% luminal narrowing at another SES site of the LCX (Fig. 1, left). The ISR lesion of the SES site at LCX was accompanied by PSS of irregular segmental type 5. The BMS site of RCA was intact. Then the patient immediately underwent revascularization for the culprit lesion of the SES sites at LAD. After thrombus aspiration, A PSS of irregular segmental type was evident also at the SES site of LAD. At the distal edge of SES site of LAD, plaque‐like projection was observed, so we placed a BMS at distal of the SES sites with overlapped edge to edge. Twenty days later, we planned target lesion revascularization for the ISR lesion of SES site at LCX. Prior to percutaneous coronary intervention, we performed observation of the each stent site by optical coherence tomography (OCT) and coronary angioscopy, especially at the PSSs sites. The coronary angiographic finding showed that the large number of PSSs was still evident at both SES sites of LAD and LCX (Fig. 1). The OCT observation for LAD showed that incomplete stent apposition (ISA) at mid‐ to distal SES site, where the PSSs were angiographically evident (Fig. 2A and B). At the SES sites, there was no lipid‐pool‐like attenuation in neointima. Coronary angioscopic finding at this site showed that neointimal coverage over the stent struts was absent and red thrombi were adhered on the naked strut. In addition, the neointima around the struts was white‐colored (Fig. 2E and F). At proximal SES site, where the PSSs were absent, both OCT and coronary angioscopic findings showed that stent struts were covered by white‐colored homogeneous neointima (Fig. 2C and G). At the site of BMS newly implanted in the LAD at admission, stent struts were naked but stent apposition was complete by both OCT and coronary angioscopic findings (Fig. 2D and H). At the SES site in LCX, where the ISR was present and the PSSs were also evident, multiple interstrut hollows (MIH) 3 were seen by the OCT observation (Fig. 3A and B). However, the stent struts between MIH were sufficiently covered with homogenous white‐colored neointima by both OCT (Fig. 3A and B) and coronary angioscopic findings (Fig. 3E and F). At the most stenotic site, stent struts were covered with thick homogenous neointima (Fig. 3C and G). At the SES site in LCX, where the PSSs were absent, the MIH was absent and the stent struts were covered by white‐colored neointima by both OCT (Fig. 3D) and coronary angioscopic findings (Fig. 3H). There was no evidence of thrombi at any SES sites of LCX. At the BMS site in the distal RCA, where the stent was implanted 13 years ago, the stent struts were covered by homogeneous high‐intensity neointima (Fig. 4).

## Discussion

The mechanism of VLST after DES implantation is assumed thrombus formation associated with incomplete neointimal coverage over the stent struts 6, 7 or with neoatherosclerosis 8. At the late stage after implantation, neointimal coverage over the stent struts are often lacking in case of DES, although struts of BMS are covered with neointima. In case of DES, moreover, even if stent is adequately implanted with a sufficient apposition, ISA, that is, a gap between struts and vessel wall occasionally develop at the late stage. These phenomena can be detected by IVUS or OCT observation. The tissue around struts was affected with apoptosis and necrosis by eluting drug 9, 10, possibly leading to formation of such a gap. The gap may yield minimal stagnation of blood flow around struts and cause stent thrombosis 11. On the other hand, the OCT can also detect MIH 3, an existence of multiple hollows between and outside well‐apposed stent struts. Both ISA and MIH are detected angiographically as PSSs. In our case, at first, we supposed also a possibility that thrombosis was caused by neointimal rupture associated with neoatherosclerosis. However, the OCT findings showed ISA at the proximal site of SES but not attenuation in neointima that represented lipid pooling in the LAD. In addition, the coronary angioscopy showed white‐colored plaque in LAD as well as LCX. Therefore, the etiology of VLST in this case was considered to be rather associated with PSSs. Noteworthily, at the PSSs site of LAD, the OCT finding showed that the ISA was evident and that the stent struts were exposed to the vessel lumen. In addition, the coronary angioscopic finding at this site showed that red thrombi were adhered on the naked strut. On the other hand, at the SES site in LCX, where the PSSs were evident but the VLST did not occur, the OCT observation demonstrated MIH but not ISA. The stent struts between MIH were sufficiently covered with homogenous neointima, and there was no evidence of thrombi at this site. From our case, we experienced different features of angiographic PSSs, that is, ISA and MIH. Also, we can envision that the development of VLST related to PSS might depend upon whether or not neointimal coverage over the stent struts.

Unfavorable outcomes after SES implantation such as loss of neointimal coverage or ISA might be associated with impaired wound healing response at the stent‐injured vessel sites, including at late‐stage relapse and prolongation of local inflammatory reaction that was strongly suppressed at acute stage after implantation 12. In contrast, new‐generation DESs such as everolimus‐eluting stent accomplishes greater strut coverage with less inflammation, less fibrin deposition and less stent thrombosis, compared to the first‐generation DESs such as SES in human autopsy analysis 13. After new‐generation DESs era, the stent thrombosis rate actually decreased, so DAPT may not need to maintain persistently. However, a recent large clinical trial demonstrated that continuation of DAPT beyond 1 year, as compared with aspirin alone, significantly reduced the risk of stent thrombosis and major adverse cardiovascular and cerebrovascular events 14. In this study, new‐generation DESs were used in 60% of patients. Therefore, even in the new‐generation DES era, aspirin monotherapy beyond 1 year after stent implantation would be questionable. Anyway, the desirable duration of DAPT after DES implantation still remains unclear. According to RESTART registry 15, in regard to VLST after SES implantation, calcified lesion, diabetes and dialysis patients require close attention and especially an interruption of DAPT suchlike in surgical therapy may be the risk independent of timing after stenting. Our patient was free from such risk factors, and already had discontinued DAPT but had been taken aspirin monotherapy. If we could detect PSSs and ISA at earlier stage and continued or resumed DAPT, the development of VLST might be prevented in this case. Although the first‐generation DESs are not used nowadays, there are many patients in whom they have been implanted. Therefore, we should do careful follow‐up for such patients also from now on, using imaging modalities such as the OCT and/or coronary angioscopy in addition to coronary angiography.

## Conclusion

We reported a case with VLST after SES implantation beyond 9 years. It was assumed that the etiology of VLST in this case was due to the late acquired ISA. Using observation by OCT and coronary angioscopy, the different features of angiographic PSSs, ISA and MIH were found in the same patient. If we had a case with angiographic PSSs after the first‐generation SES, we need a further observation using imaging modalities such as OCT and/or coronary angioscopy to evaluate the risk of VLST due to PSSs and to continue or to resume the DAPT if necessary.

## Conflict of Interest

None declared.

## Authorship

MK and SA: managed the patient's course and wrote the primal draft. IK, HN, SK, TN, SY and MS: aided in manuscript editing and also contributed to the literature searching. TI: contributed substantially to make the design of this report and also approved the final version of the manuscript.
